# Cellular interactions in tumor microenvironment during breast cancer progression: new frontiers and implications for novel therapeutics

**DOI:** 10.3389/fimmu.2024.1302587

**Published:** 2024-03-12

**Authors:** Tosin Akinsipe, Rania Mohamedelhassan, Ayuba Akinpelu, Satyanarayana R. Pondugula, Panagiotis Mistriotis, L. Adriana Avila, Amol Suryawanshi

**Affiliations:** ^1^ Department of Biological Sciences, College of Science and Mathematics, Auburn University, Auburn, AL, United States; ^2^ Department of Chemical Engineering, College of Engineering, Auburn University, Auburn, AL, United States; ^3^ Department of Anatomy, Physiology, and Pharmacology, College of Veterinary Medicine, Auburn University, Auburn, AL, United States; ^4^ Department of Pathobiology, College of Veterinary Medicine, Auburn University, Auburn, AL, United States

**Keywords:** tumor microenvironment, stroma, immune cells, breast cancer, metastasis

## Abstract

The breast cancer tumor microenvironment (TME) is dynamic, with various immune and non-immune cells interacting to regulate tumor progression and anti-tumor immunity. It is now evident that the cells within the TME significantly contribute to breast cancer progression and resistance to various conventional and newly developed anti-tumor therapies. Both immune and non-immune cells in the TME play critical roles in tumor onset, uncontrolled proliferation, metastasis, immune evasion, and resistance to anti-tumor therapies. Consequently, molecular and cellular components of breast TME have emerged as promising therapeutic targets for developing novel treatments. The breast TME primarily comprises cancer cells, stromal cells, vasculature, and infiltrating immune cells. Currently, numerous clinical trials targeting specific TME components of breast cancer are underway. However, the complexity of the TME and its impact on the evasion of anti-tumor immunity necessitate further research to develop novel and improved breast cancer therapies. The multifaceted nature of breast TME cells arises from their phenotypic and functional plasticity, which endows them with both pro and anti-tumor roles during tumor progression. In this review, we discuss current understanding and recent advances in the pro and anti-tumoral functions of TME cells and their implications for developing safe and effective therapies to control breast cancer progress.

## Graphical Abstract

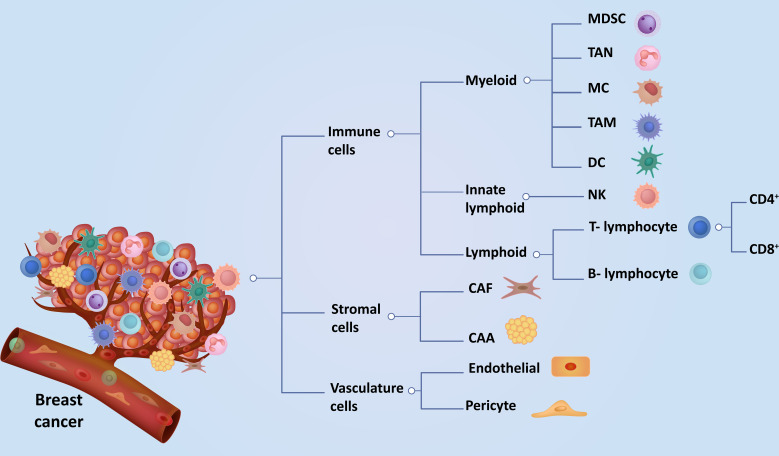

## Introduction

Breast cancer is the most common and frequently diagnosed cancer in women worldwide, with more than 2 million new breast cancer cases reported annually (1). Globally, breast cancer is the leading cause of cancer deaths in women (2, 3). Based on gene profiling, breast cancer can be classified into five molecular subtypes - Luminal A, Luminal B, Human epidermal growth factor receptor 2 (HER2), Basal-like, and Triple-negative breast cancer (TNBC) (4). Although these classifications are not static, ongoing research may lead to refinements or the discovery of new subtypes. Molecular classification plays a crucial role in tailoring treatment strategies for breast cancer patients, helping oncologists choose the most effective therapies based on the specific molecular characteristics of the tumor (5). Additionally, the TNM staging system proficiently evaluates patients by effectively assessing the extent of the tumor (T), involvement of lymph nodes (N), and presence of metastasis (M). Due to differences in molecular characteristics of breast cancer sub-types, the use of biomarkers, histologic grade, HER2 expression, hormone receptor, and multigene panels have now been incorporated into the conventional TNM staging (6). The tumor microenvironment (TME) of breast cancer plays a central role in tumor progression, immune evasion, and resistance to conventional anti-cancer therapy (7). Breast TME mainly comprises cancer, immune, and stroma cells. Apart from cancer cells, the cellular components of breast TME can be broadly classified as immune cells (myeloid, innate lymphoid, and lymphocytes), stromal cells (fibroblasts and adipocytes), and vasculature cells (endothelial cells and pericytes) (**Graphical Abstract**). The various cellular components of breast TME exhibit intricate and dynamic interactions that significantly impact cancer progression, metastasis, immunosuppression, and resistance to both conventional and emerging immunotherapies (8, 9). The complex molecular and cellular interplay among the TME constituents provides essential nutrients, oxygen, and growth factors that facilitate efficient tumor cell proliferation and progression (10, 11). The surrounding stroma’s cellular, genetic, structural, functional, and epigenetic alterations profoundly impact the plasticity and morphogenesis of epithelial cells, thereby contributing to tumorigenesis (12). Recent breakthroughs and extensive studies from preclinical studies (**Table 1**) and clinical trials (**Table 2**) have indicated that alterations in breast TME signatures can serve as valuable prognostic indicators and aid in the development of innovative anti-cancer therapies (27). Consequently, there has been a notable shift towards targeting the key components of the TME in the development of novel treatments (27, 28). In this review, we discuss the current understanding of cancer, stromal, vasculature, and immune cell interactions within the breast TME and their implications for developing novel, safe, and effective breast cancer treatments.

**Table 1:** Selected pre-clinical studies showing the suppression of tumor progression by targeting TME-associated cells and effector molecules.

Model	Agent	Target	Antitumor Effect	Ref.
BALB/c mice	Radiotherapy	CXCL16, a chemokine that binds to CXCR6 on Th1 and activated CD8 effector T cells	Increased the migration of CD8**^+^** CXCR6**^+^** activated T cells to tumors	(13)
67NR mouse	Combination therapy (Radiotherapy + Immunotherapy)	Immune checkpoints CTLA-4 and PD-L1	Radiation in combination with anti-CTLA-4 and/or anti-PD-L1 blockade stimulates CD8**^+^** T cell-mediated anti-tumor immunity	(14)
4T1 mouse	Combination therapy (Immunotherapy + chemotherapy)	Immune checkpoint	Suppression of MDSCs leads to regression of tumor cells	(15)
BALB/c mice	Administration of the Toll-like receptor (TLR) 7/8 agonist 3M-052	TLR 7/8	Enhances interferon-driven tumor immunogenicity and suppresses metastatic spread in preclinical triple-negative breast cancer	(16)
MDA-MB-231 xenograft	Knockdown of lysyl oxidase (LOX) β-aminopropionitrile (BAPN), miRNA-142-3p	LOX inhibition	Overcome chemoresistance in TNBC	(17)
4T1 tumor-bearing mice	Doxorubicin	DC, CD44^+^ Cancer stem cells	Immunotherapy	(18)
Mammary tumor-bearing mice	Macrophage recruitment blockade + Paclitaxel	TAM	Reprogram the TME to decrease primary tumor progression, reduce metastasis, and improves survival by CD8**^+^** T-cell–dependent mechanisms.	(19)
4T1-Neu mammary tumor-bearing mice	Docetaxel	MDSC	Docetaxel treatment polarized MDSCs toward an M1-like phenotype	(20)
MDA-MB-231 xenograft	Eribulin	TME vasculature	Vascular remodeling: Improved perfusion Increased microvessel density. Decreased mean vascular areas. Fewer branched vessels in tumor tissues,	(21)
MCF-7 xenograft	Paclitaxel	CAF	Improved local drug accumulation	(22)
BALB/c Neu-transgenic mouse	Local delivery of IL-21	TAM	Identified that abundant TAMs are a major extrinsic barrier for anti-Her2/neu Ab therapy and present a novel approach to combat this extrinsic resistance to skew TAM polarization from M2 to M1	(23)
MCF7 breast cancer cells	B7-H3 Knock-down	DC (B7-H3/CD 276)	Inhibit the proliferation of CD4**^+^** and CD8**^+^** T cells. Inhibit the release of IFN-γ by decreasing mTOR signaling	(24)
KBP-mice	PD-L1 inhibitors	PD-L1	PD-L1 blockade reverts the expression of PD-L1 in macrophages and synergizes with paclitaxel to reduce tumor growth in TNBC	(25)
C57BL/6 mice and nude mice	5-Fluorouracil (5FU)	MDSC	5FU selectively induced MDSC apoptotic cell death leading to IFN-γ production by tumor-specific CD8**^+^** T cells infiltrating the tumor and promoting T cell-dependent antitumor responses in vivo	(26)
MDA-MB-231 xenograft	Capecitabine + Eribulin	TME vasculature	Decreased hypoxia-associated protein expression of VEGF	(21)

**Table 2:** Selected clinical trials on breast TME-related targeting modalities (https://www.clinicaltrials.gov/).

Strategy/Rationale	Condition	Intervention	Identifier	Outcome measures	Status/ Stage
To quantify CD4 and CD8 in other to identify biomarker changes in the immune microenvironment induced by neoadjuvant chemotherapy	TNBC	Analysis of a list of biomarkers before and after sequential treatment with FEC100 or EC100 then taxane, and paclitaxel weekly	NCT04368468	Identifying biomarkers present in the residual disease would be a criterion to guide the choice of post-neoadjuvant adjuvant systemic treatment, so as to personalize it.	Completed
To evaluate the effects of orally administered reparixin on the TME, cancer stem cell (CSC) markers, and cytokine inflammation markers	HER2- metastatic breast cancer	Fixed dosage of Paclitaxel+ three increasing dosage of Reparixin were used	NCT02001974	Explores the safe dose limit in treating MUC1-positive advanced breast cancer	Phase 1
To evaluate the role of soluble immune checkpoints in predicting the response to neoadjuvant therapy	Breast cancer	Immune checkpoint measurement	NCT05519397	Measurement of sCD25 (IL-2Ra), 4-1BB, B7.2 (CD86), Free Active TGF-β1, CTLA-4, PD-L1, PD-1, Tim-3, LAG-3, Galectin-9	Completed
To differentially compare the breast TME between Luminal A and TNBC with and without Radiation Treatment	Luminal A and TNBC	The mean percent change in TILs in tumor tissue from initial core biopsy samples will be compared with pathology samples from definitive surgery after irradiation between the two different breast cancer sub-types	NCT03165487	Identifying these differences in proteins may allow them to be used in the future as markers to predict the likelihood of tumors recurring.	Recruiting
To determine the clinical response in patients with HER2/neu-positive stage I-III breast cancer and bone marrow micrometastases treated with the drugs of interest	HER2/neu-positive	Bevacizumab, Trastuzumab, Carboplatin, Docetaxel	NCT00949247	To study how well giving docetaxel and carboplatin together with trastuzumab and bevacizumab works in treating patients with stage I, stage II, or stage III breast cancer and bone marrow micrometastases.	Early Phase 1
To evaluate the T Cell response to a peptide-based vaccine in patients with breast cancer	Breast cancer	Biological: 9 Peptides from Her-2/neu, CEA, & CTA	NCT00892567	To study the concentrations of Persistent Organics Pollutants in both adipose tissue and serum samples from breast tumor patients	Phase 1
To evaluate the impact of single dose versus three doses of Stereotactic Radiation Therapy (SBRT) prior to surgery	Early-stage breast carcinoma	Radiation: Stereotactic body radiation followed by lumpectomy	NCT02212860	Immune priming: (Quantify TILs, PDL-1, neutrophils, and macrophages) Measure angiogenesis (VEGF), proliferation (Ki67), hypoxia (HIF1/HIF2), and invasion (SDF-1) markers	Completed
To evaluate the effects of MK-3475 (Pembrolizumab) on the breast tumor microenvironment	TNBC	Merck 3475 Pembrolizumab	NCT02977468	To determine if immune modulation therapy with MK-3475 will increase TILs in newly diagnosed TNBC tumors will alter the expression of immune tolerant markers [including PD-L1], within the primary tumor.	Phase 1
To evaluate the effect of Palbociclib plus Letrozole in Hormone receptor-positive residual disease after neoadjuvant chemotherapy	Hormone Receptor (HR) Positive/ HER2 Negative	Palbociclib, Letrozole	NCT04130152	Study the changes in TILs and PDL-1 following treatment with palbociclib plus letrozole, after neoadjuvant chemotherapy	Early Phase 1
To comparatively evaluate the efficacy, safety, and pharmacokinetics of atezolizumab (MPDL3280A) administered with nab-paclitaxel	TNBC	Atezolizumab (an anti-PDL1 antibody) + Nab-Paclitaxel	NCT02425891	Atezolizumab plus nab-paclitaxel prolonged progression-free survival among metastatic TNBC in the intention-to-treat population and the PD-L1–positive subgroup.	Phase 3

## Breast TME

**Figure 1:**
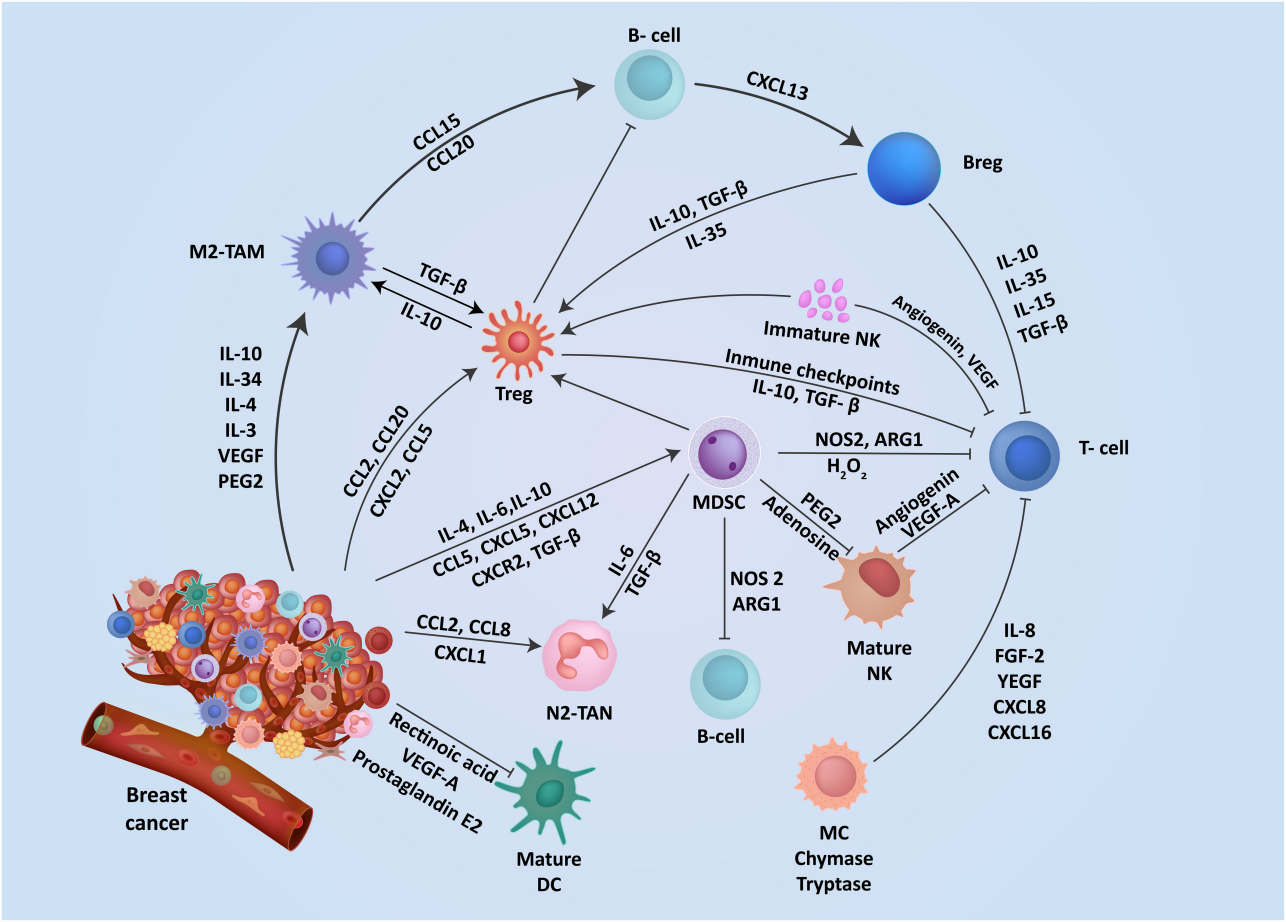
The interplay of mediators aid immunosuppression in breast TME. The TME contains a range of resident cells playing a key role in the progression and metastasis of breast cancer cells. These resident cells and their associated secretory elements and receptors including cytokines, chemokines, and stimulatory growth factors are shown. Cells in the TME exhibit a diverse network of mediators that actively engage in promoting an immunosuppressive TME.

**Figure 2:**
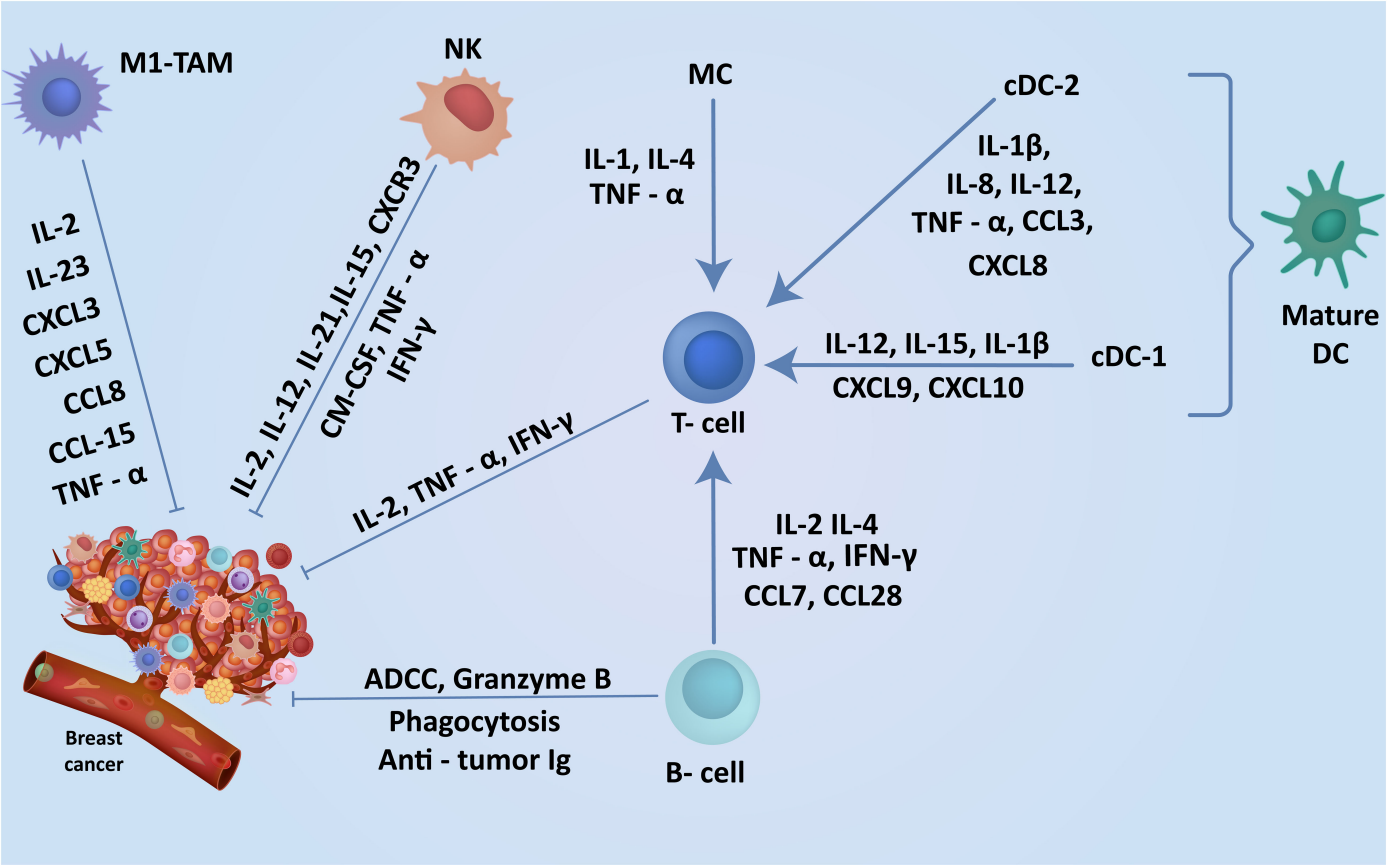
Cells in breast TME regulate the induction of robust anti-tumor immunity. The TME contains a range of anti-tumor cells including TILs, DCs and macrophages in the breast playing a key role in the breast cancer suppression. The expression of these cells within the breast cancer TME and understanding their anti-tumor function may enhance the discovery of new markers associated with specific subtypes leading to earlier diagnosis and better clinical outcomes.

Breast TME is highly plastic and undergoes constant changes and stage-specific adaptations depending on numerous cancer cell-intrinsic and extrinsic factors. These alterations in the TME are characterized by networks of cytokines and growth factors, disrupted signaling pathways, and modified molecular signatures in the stroma (29). Extensive research on TME characterization has highlighted the crucial role of communication between tumor cells and stroma in driving breast cancer oncogenesis, progression, and metastasis (**Figure 1**) (30, 31). The breast tumor stroma comprises various components, including fibroblasts, immune cells, endothelial cells, adipocytes, and pericytes (32). Throughout the progression of breast cancer, the stroma undergoes significant changes, including the formation of cancer-associated fibroblasts (CAFs), infiltration of immune cells, inflammation, angiogenesis, and remodeling of the extracellular matrix (ECM) (33, 34). These alterations disrupt the integrity of the basement membrane, facilitating the spread of tumor epithelial cells into the stroma (35). Since these various molecular and cellular components have a direct influence on breast cancer progression, they represent attractive targets for therapeutic development. The immune cells within the breast TME play a critical and dynamic role in cancer progression and anti-tumor immunity (36). Most immune cells are plastic in their functional phenotype and can adapt in response to local TME factors, allowing them to play dual pro or anti-tumor roles (37). Effector immune cells infiltrating the TME can directly eliminate neoplastic cells expressing neo-antigens on their surface and suppress tumor progression (**Figure 2**) (38). However, tumors employ numerous immune evasion strategies to impede immune cell infiltration and hinder their effector functions within the TME (37). The immune cell repertoire within the breast TME can be broadly classified as myeloid, innate lymphoid, and lymphoid cells. Myeloid cells include myeloid-derived suppressor cells (MDSCs), tumor-associated neutrophils (TANs), tumor-associated macrophages (TAMs), dendritic cells (DCs), mast cells (MCs), etc. Natural killer (NK) are innate lymphoid cells with cytotoxic effector functions and play a crucial role in anti-tumor immunity. Lymphoid cells include B lymphocytes and numerous subsets of T-lymphocytes that play a central role in tumor-antigens-specific anti-tumor immunity (39).

In subsequent sections, we will briefly discuss the interplay of immune and non-immune (stromal and vasculature) cells and how these complex interactions can be strategically targeted to develop novel, safe, and highly effective therapies for breast cancer patients.

### Myeloid cells in breast TME

#### Myeloid-derived suppressor cells

Myeloid cells, derived from hematopoietic stem cells in the bone marrow, play a crucial role in initiating innate and adaptive immune responses (40). However, these cells undergo impaired differentiation during cancer progression, resulting in immature phenotypes with reduced phagocytic capacity and immunosuppressive function (41). MDSCs are prominent cell types in the breast TME that rapidly proliferate and promote tumor progression, angiogenesis, and metastases (42, 43). When activated, MDSCs contribute to immunosuppression and cancer invasiveness through increased production of reactive nitrogen species (RNS), reactive oxygen species (ROS), and arginase 1 (ARG1) expression (44, 45). Human MDSCs in the bloodstream can be classified into two types: granulocytic MDSCs (G-MDSCs) and monocytic MDSCs (M-MDSCs) (46). G-MDSCs are further categorized based on cell surface marker expression as CD11b^+^CD14^-^CD66^+^ and CD11b^+^CD14^-^CD15^+^. Similarly, M-MDSCs are characterized by the cell surface markers CD11b^+^CD14^+^CD15^-^ (47). MDSCs exhibit low expression of Human Leukocyte Antigen–DR isotype (HLA-DR) and CD14, the cell surface receptors essential for proper immune responses to antigens, resulting in an immune response defect (43, 48). The activation and recruitment of MDSCs in the TME is mediated through increased production of specific chemokines, cytokines, and factors, including IL-4, IL-6, IL-10, IL-12, IL-13, CCL5, CCL2, CXCL2, CXCL5, CXCL12, vascular endothelial growth factor (VEGF)-A, transforming growth factor (TGF)-β, and granulocytic-colony stimulating factor (G-CSF) in TME (49–52). These molecules are critical in shaping the tumor microenvironment and promoting MDSC-mediated immune suppression. MDSCs have been found to play a crucial role in the immunosuppressive microenvironment by facilitating the development of CD4^+^Foxp3^+^ regulatory T (Treg) cells and promoting immunosuppressive phenotype in macrophages (53, 54). Additionally, MDSCs express CD40, increasing Treg-mediated tumor immune tolerance (55). CD40, a member of the tumor necrotic factor (TNF) receptor superfamily, is expressed on antigen-presenting cells (APCs), while its ligand (CD40L) is primarily expressed on activated T and B cells (56). The interaction between CD40 and CD40L promotes the development of adaptive immunity (57). When exposed to increased stimulation by IFN-γ, G-MDSCs upregulate the expression of CD40 and MHC II, leading to the induction of Tregs and the suppression of T cell proliferation (55). MDSCs also contribute to angiogenesis, maintain cancer stem cells (CSCs), and inhibit CD8^+^ T cell activation through the expression of nitric oxide synthase 2 (NOS2) and ARG1 (58, 59). Using microarray analysis, Hix et al. compared the low-aggressive TM40D and highly aggressive TM40D-MD mouse mammary carcinoma cells and discovered a positive correlation between tumor-recruited CD33^+^ myeloid cells and the progression of human breast cancer from DCIS to IDC (60). Additionally, they found a significant association between CD33^+^ MDSCs and poor prognosis and worsened overall survival (OS) in the ER^-^ subtype (61). Furthermore, the transcriptional factor deltaNp63 enhanced the recruitment of MDSCs and correlated with poor prognosis and metastasis in TNBC (62). A pre-clinical study revealed the major role of CXCR2^+^ MDSCs, a subtype of MDSCs, in breast cancer metastases (63). Moreover, MSDCs indirectly regulate immune response and hinder cancer immunotherapy by interacting with other components of the TME (64). The critical role of TME MDSCs in causing immunosuppression and resistance to cancer immunotherapies during breast cancer progression underscores the need for further comprehensive studies to successfully develop innovative immunotherapies.

#### Tumor-associated neutrophils

Neutrophils, comprising 50-70% of circulating leukocytes, represent the body’s primary defense against infections (65). Additionally, they play a crucial role in tumor progression by infiltrating the TME. The TME regulates the recruitment and polarization of neutrophils, allowing them to develop either an anti-tumor (N1) or pro-tumor (N2) phenotype in response to cytokines present in the TME (66, 67). N1 polarized neutrophils exhibit a robust immune profile characterized by elevated levels of TNF-α, CCL3, ICAM-1, and reduced arginase expression. On the other hand, N2 TANs overexpress several chemokines, including CCL2, CCL8, CXCL1, CXCL2, etc (68). The increased NADPH oxidase activity of N1-like neutrophils leads to the generation of cytotoxic ROS, which can effectively target tumor cells (69). Despite the critical role of neutrophils in the TME (70, 71), further studies are warranted to investigate molecular and cellular networks that drive immunosuppressive phenotype in neutrophils in TME. Studies have demonstrated the preferential migration of neutrophils into specific breast tumor subtypes, such as hormonal negative ductal adenocarcinoma and TNBC (72). Additionally, TGF-β has been shown to promote the N2 phenotype in neutrophils infiltrating the TME (66). TANs in the TNBC TME are a source of proangiogenic factors and matrix metalloproteinase (MMP)-9, a protease crucial in ECM remodeling (73). MMPs and gelatinase B/MMP-9 actively degrade the extracellular matrix, promoting tumor invasiveness and metastasis (74, 75). MMP-9 also contributes to angiogenesis and tumor progression by releasing VEGF-A and inhibiting anti-angiogenic molecules (70). TANs’ inability to express tissue inhibitors of metalloproteinase-1 (TIMP-1) enhances the angiogenic potential of neutrophil-derived MMP-9 in the TME, unlike cells expressing the MMP-9/TIMP-1 complexes (76). Recent findings have shown that in the presence of CD90, TIMP-1 expressed by TANs induces epithelial-mesenchymal transition (EMT) in breast cancer, facilitating metastasis (77). As a result, a significant reduction in the spread of cancer has been observed through CD90 blockade (77). Several strategies can be employed to target TANs, including preventing neutrophil migration to tumors, hindering their polarization into N2-type, and targeting neutrophil-associated mediators (71, 78). However, further studies are warranted to characterize TANs’ pro-tumoral role during breast cancer progression properly.

#### Mast cells

MCs demonstrate a vital role in both innate and adaptive immunity. Positioned within epithelial and mucosal tissues throughout the body, MCs effectively regulate various immune and non-immune cell types, including T and B lymphocytes, endothelial cells, fibroblasts, macrophages, and DCs (79). Notably, MCs exhibit a dual function in breast cancer progression (80). Their ability to produce anti-tumoral cytokines, such as IL-1, IL-4, IL-6, and TNF-α facilitates CD8^+^ priming and maturation (81). Conversely, MCs can assume pro-tumor roles by increasing the production of immunoregulatory molecules, including IL-8, fibroblast growth factor (FGF)-2, TGF-β, VEGF-A, CXCL8, and CXCL16 (82). Such effector molecules released by MCs hinder immunity, degrade the ECM, and enhance tumor vascularization, thus modifying the TME (82, 83).

In the context of breast TME, MCs actively promote cell proliferation, invasiveness, and metastases, ultimately correlating with a poor prognosis (84). Additionally, MCs are crucial in promoting angiogenesis through secretion of angiogenic cytokines (85). MC stabilizer, disodium cromolyn, has demonstrated its ability to induce an anti-tumor effect by effectively inhibiting the production of VEGF and platelet derived growth factor (PDGF) (86). The infiltration of human MC subpopulations within the TME can be classified based on their expression of the proteases chymase, tryptase, or tryptase-chymase (87). The involvement of chymase and tryptase in ECM remodeling and the production of angiogenic factors highlights their significant role in promoting invasiveness (88). The functions of tryptase and chymase MCs in breast cancer are specific to subtypes. Research by Glajcar et al. revealed a significantly higher presence of the MC tryptase-chymase subset in luminal A and B tumors compared to HER2^+^ and TNBC, indicating relevance in these subtypes (89). Various studies have also shown the contribution of tryptase^+^ MCs to tumor progression in TNBC and luminal A breast cancer (89, 90). Although MC stabilizers and protease inhibitors have been successfully used in other cancers, their clinical effectiveness in breast cancer remains uncertain (91). Conversely, a recent report suggested the increased infiltration of MCs is associated with lower tumor grade, reduced tumor proliferation, and decreased HER2 overexpression (92). Further studies are needed to fully comprehend MC function and explore their potential as therapeutic targets in breast cancer.

#### Tumor-associated macrophages

TAMs are abundant immune cells within the TME (93). Human blood monocytes undergo differentiation into naïve macrophages (M0) and subsequent polarization into M1 and M2 phenotypes mediated by IFN-γ and IL-4, respectively (94, 95). M1 macrophages are highly phagocytic and are associated with CD4^+^ polarization towards IFN-γ producing Th1 cells (95). M1-like macrophages possess the capability to induce acute inflammatory responses through the production of inflammatory cytokines and chemokines such as IL-2, IL-12, IL-23, TNF-α, CXCL3, CXCL 5, CCL8, CCL15, as well as reactive nitrogen and oxygen intermediates, which exert antitumor effects (96, 97). Resident macrophages play a critical role in host defense (98). However, macrophage populations in TME adapt to an anti-inflammatory, M2-like phenotype (99). The recruitment of TAMs to the TME is promoted by stromal and tumor cells’ production of chemokines and growth factors (100). Peripheral blood monocytes derived from the bone marrow are recruited to the tumor site and undergo differentiation into TAMs (101). The CSF is an integral factor in regulating the recruitment of macrophage populations (102). The recruitment of peripheral blood monocytes to the tumor site is facilitated through chemokine receptors expressed on monocytes and chemokine gradient in TME. One such example is the binding of CCL2 to CCR2 and CCR5 receptors on monocytes, leading to monocyte recruitment to the TME (103). Another example is the binding of CCL20 to CCR6 receptors (104).

In breast cancer, the polarization of monocytes to TAMs is influenced by various factors, including tumor-derived factors produced by breast cancer cells and other cells in the TME (105). Monocyte differentiation into TAMs is mediated by VEGF-A and IL-4 (106). M2 TAM differentiation can occur through IL-4 secreted from Th2 cells and IL-10 derived from Tregs (107). IL-10 inhibits the production of pro-inflammatory chemokines by macrophages and promotes the self-polarization of TAMs (108). Furthermore, alternative M2 activation of TAMs is elicited by IL-34 and IL-13 derived from Th2 cells, eosinophils, or basophils (102). These macrophages serve as a significant source of proteolytic enzymes that facilitate the destruction of the ECM and promote neoplastic cell invasion (74).

TAMs contribute to immune evasion by producing IL-10, EGF, and TGFβ (99, 109). The EGF produced by TAMs actively stimulates the proliferation of breast carcinoma cells (110), whereas TAM-produced IL-10 promotes the accumulation of tumor cells at distant sites (111). Furthermore, TGFβ originating from TAMs enables monocyte efflux (112). Also, TAMs facilitate tumor cell growth, angiogenesis, metastasis, and immune evasion by recruiting Tregs (113). TAMs can also establish cancer stem-cell niches, leading to tumor chemotherapy resistance (114). In TNBC, TAMs consistently activate hepatic leukemia factor (HLF) through the IL-6-TGF-β1 axis. HLF transactivates gamma-glutamyltransferase 1 (GGT1), which promotes ferroptosis and cisplatin resistance, ultimately driving malignancy in tumor cells (115). High infiltration of TAMs is associated with a worsened prognosis in breast cancer patients (116). TAMs and DCs play a pivotal role in inducing and regulating effector T cell and Treg responses within the TME, thereby influencing resistance to recently developed immune-checkpoint blockade (ICB) therapies (93). The Wnt/β-catenin pathway is critical for several biological processes (117). However, its dysregulation has been associated with the development of cancer and other diseases. TAMs and DCs activate the Wnt/β-catenin pathway to induce immune tolerance, inhibiting effector T-cell responses and promoting regulatory T-cell responses (118). Consequently, targeting the Wnt/β-catenin pathway holds promise for effective therapeutic interventions in breast cancer (119). Research on TAMs has led to the development of macrophage-focused treatment approaches, which are currently undergoing clinical trials for breast cancer (120). These strategies involve suppressing macrophage recruitment, reprogramming TAMs towards an anti-tumor phenotype, and enhancing macrophage-mediated phagocytosis or tumor cell killing (100, 116).

#### Dendritic cells

DCs are critical in maintaining immune surveillance and achieving a delicate equilibrium between protective immunity and immune tolerance (121). However, tumors exploit these mechanisms to regulate anti-tumor immunity (122). DCs can be categorized into various subsets based on their location, phenotype, and antigen presentation abilities (123). As professional APCs, DCs are pivotal in initiating and activating anti-tumor T cell responses in tumor-draining lymph nodes and the TME (124). During breast cancer progression, DCs engage in phagocytosis of apoptotic tumor cells, process and present tumor antigens on MHC-I and MHC-II molecules, migrate to local lymph nodes, and present antigens to naive CD4^+^ and CD8^+^ T cells to elicit an anti-tumor immune response (125, 126). Additionally, DCs in the TME secrete chemokines and cytokines that play a crucial role in recruiting and activating effector CD4^+^ and cytotoxic CD8^+^ T cells for effective anti-tumor immune responses (127).

Transcriptional profiling has identified specific subsets of DCs in both normal breast tissue and breast TME (128). While the nomenclature and classification of DCs in the TME can be complex, DCs in TME can be broadly classified into three subsets, including plasmacytoid DCs (pDCs), monocytic DCs (moDCs), and conventional DCs (cDCs), which are further classified as cDC-1 and cDC-2 (129, 130). pDCs play a significant role in cross-presenting tumor antigens on MHC-I molecules to initiate cytotoxic CD8^+^ T Lymphocytes (CTL)-mediated anti-tumor responses (131). pDCs also secrete large amounts of type I interferons (IFN-α/β) (131). Recent studies have shown that pDCs in breast TME promote Treg responses, negatively impacting prognosis and survival rates (132, 133). Additionally, gene expression analysis has revealed that pDC-related genes are among the top genes associated with an increased risk of breast cancer metastasis (134). However, other studies have contradicted these findings, highlighting a better prognosis and increased survival linked to pDCs (135–137). Circulating monocytes can differentiate into moDCs within the TME and are primarily responsible for inducing CD4^+^ T cell-mediated responses (138). However, the immunosuppressive TME often leads to a tolerogenic phenotype in moDCs, increasing pro-tumor Treg responses (139). cDC-1 and cDC-2 in the TME play a critical role in capturing tumor antigens to activate CD8^+^ and CD4^+^ effector T-cell responses (134). cDC-1 can be identified by the expression of markers such as IRF8, BDCA3, BATF3, CLEC9A, and CD103 (140). They produce cytokines (IL-12, IL-15, IFN-β) and chemokines (CXCL9 and CXCL10) to mount a robust immune response (128). In the luminal and TNBC subtypes, cDC-1 has been associated with improved disease-free survival (DFS) and positive patient outcomes through its activation and expansion of CD103, enhancing the tumor response to therapeutic programmed death-ligand 1 (PD-L1) and BRAF inhibition (141, 142). cDC-2 express various markers such as IRF4, CD11b, SIRPα, CLEC10A, and CD1C, and produce IL-1β, IL-6, IL-8, IL-12, TFN-α, CCL3, and CXCL8 to activate anti-tumor T cell responses (143, 144).

DCs have been found to exhibit a dual pro-tumoral and anti-tumoral role depending on the cytokine milieu in the TME and their maturation state (145, 146). For instance, immature DCs support angiogenesis in rapidly growing angiogenic tumors, while mature DCs suppress angiogenic characteristics (147). Additionally, infiltration of mature DCs in primary tumors is associated with reduced metastasis and improved clinical outcomes (148). The increased expression of CD83, a marker for mature DCs, is strongly linked to improved survival in node-positive tumor patients, particularly in TNBC patients with mature CD11c^+^ (149, 150). Moreover, the presence of CD83^+^ in the peri-tumoral region of IDC lesions suggests a potential role for mature DCs, while immature CD1a^+^ DCs are found at tumor edges (151). Previous studies indicate that the immature DC phenotype promotes primary tumor progression to IDC (148, 152). Furthermore, elevated levels of pro-tumor molecules like VEGF-A and prostaglandin E2 in the TME hinder DC maturation, thereby inhibiting T-cell proliferation (153, 154). In response, therapeutic strategies targeting VEGF-A, such as anti-VEGF-A antibodies like bevacizumab, have shown promise in promoting T cell and DC infiltration in TNBC (155). Extensive research has unveiled the pivotal role played by Wnt/β-catenin signaling in the advancement of breast cancer, spanning both tumor cells and immune cells (156). The upregulation of Wnt ligands triggers the activation of canonical β-catenin signaling in DCs, thereby facilitating the generation of IL-10, TGF-β, and retinoic acid (RA) synthesizing enzymes (122, 157). This augmented production of immunoregulatory molecules by DCs within the TME fosters the development of Treg responses, overshadowing Th1 and CTLs (156, 158). These findings underscore the potential of targeting DCs in breast cancer progression as a viable therapeutic strategy, capable of stimulating robust anti-tumor immunity and suppressing regulatory T-cell responses.

### Innate lymphoid cells in breast TME

#### Natural killers

NKs are innate lymphoid cells that can directly eliminate tumor cells by releasing anti-tumor cytokines and cytolytic granules (159). Their development primarily occurs from hematopoietic stem cells (HSCs) in the bone marrow, although other origin sites, such as the thymus and liver, have been proposed (160). Essential transcriptional factors for NK cell precursors include Nfil3, Id2, and Tcf1, while maturation relies heavily on Smad4, Tox, Eomes, Gata3, T-bet, and Runx3 (161–163). Cytokines also play a crucial role in NK cell development and maturation (164–166). For example, IL-7 is responsible for generating CD122^+^ NK progenitors from HSCs, while IL-15 is critical for the development of NKs from CD122^+^ NK progenitors into mature NKs (mNKs) (167, 168). Additionally, IL-17 modulates the activities of IL-15 (166). Functionally, mNKs can be differentiated into two major subtypes: CD56^bright^CD16^dim^ NK subtypes, which make up approximately 90% and are involved in cytotoxicity, and CD56^dim^CD16^bright^ NK subtypes, which make up the remaining 10% and are responsible for antibody-dependent cell-mediated cytotoxicity (ADCC) (169). Unlike T-lymphocytes, NK cells recognize target cells expressing aberrant cell surface proteins, such as virus-infected or tumor cells, through their Fc receptors (170). The binding of NK cell Fc receptors to antibody-coated target cells leads to targeted killing through ADCC. Moreover, NK cells can eradicate cells that lack or display diminished MHC class I molecules on their cell surface, a common strategy that cancer cells employ to avoid CTL responses (171).

Tumor-infiltrating NK cells engage in immunosurveillance using a combination of activating and inhibitory receptors, effectively identifying and eliminating target cells while sparing healthy ones (172, 173). This process is facilitated by the production of various cytokines and chemokines, including TNF-α, IFN-γ, IL-2, IL-12, IL-21, IL-15, IL-18, CXCR3, and granulocyte-macrophage colony-stimulating factor (GM-CSF), which actively promote anti-tumor immunity (174, 175). Additionally, the receptors on NK cells can selectively target tumor cells by recognizing growth factors like PDGF, thereby triggering the release of IFN-γ and TNF-α to inhibit tumor growth (176). Although NK cells exhibit anti-tumor capabilities, they can also produce immunosuppressive cytokines that hinder anti-tumor immunity. NK cells secrete angiogenic factors like VEGF-A and angiogenin, which contribute to the progression of breast cancer (177). A recent study uncovered a new mechanism of cancer immune evasion, which involves inhibiting NK cells’ cytotoxic granule machinery by chitinase-3-like protein 1 (CHI3L1) (178). This protein, synthesized by tumor cells, plays a significant role in inflammation, tissue injury, and remodeling responses (179). Analysis conducted *in vitro* revealed elevated levels of CHI3L1 in the sera of trastuzumab-resistant patients compared to responders (178). CHI3L1 inhibits NK cell cytotoxicity and ADCC by disrupting the cytotoxic machinery, preventing lytic granule polarization to the immune synapse, and hindering downstream JNK signaling, a crucial process for cancer cell apoptosis (180). Furthermore, administering CHI3L1 *in vivo* weakens the control of NK cell-sensitive tumors while blocking CHI3L1 in conjunction with ADCC effectively treats HER2^+^ xenografts in mice (178).

NK cell exhaustion has been observed in an immunosuppressive TME and characterized by reduced activating receptors, decreased production of effector cytokines (181), impaired signaling/transcriptional pathways, hypoxia (182), low pH (183), upregulation of inhibitory receptors like NKG2A, TIM-3, PD-1, TIGIT, LAG-3, KIR (184, 185), and the presence of Tregs (186), Bregs (187), and MDSCs (188). This NK cell exhaustion phenotype presents a significant obstacle to developing NK cell-targeting immunotherapies. However, new strategies are being developed to combat NK cell exhaustion and enhance their anti-tumor function. For example, IL-21 treatment increases IFN-γ and granzyme B levels through Tim-3^+^PD-1^+^NK cells, reversing NK cell exhaustion (189). This highlights the potential therapeutic approach of using IL-21 to restore NK cell immunity function (190). In addition, IL-15 plays a crucial role in NK cell proliferation and survival (191). However, repetitive exposure to IL-15 during cancer treatment can diminish viable cell cycle signaling, decreased tumor control, and reduced fatty acid oxidation, resulting in NK cell exhaustion (192–194). Alternatively, an immunotherapy with membrane-bound IL-15 (mbIL15) is proposed (193, 195). By linking the human IL-15 gene to the CD8α transmembrane domain gene, mbIL15 can be created. NK cells expressing mbIL15 have been shown to activate cell cycle signaling and exhibit higher cytotoxicity against leukemia, lymphoma, and sarcoma *in vitro* and *in vivo* mouse xenograft tumor models (193). Expression profiling of NK cells can help identify dysfunction and exhaustion markers relevant to each breast cancer subtype. However, further studies on NK cell exhaustion in breast cancer are necessary.

Moreover, TME has demonstrated the capacity to modify the functionality and phenotype of NK cells (196). In a recent study by Mamessier et al., the dysfunctional tendencies of tumor-infiltrating NK cells in invasive and non-invasive breast cancer were characterized (197). Their findings unveiled a gradual reduction in the expression of NK cell activating receptors, such as NKp30, NKG2D, DNAM-1, CD16, CD226, and 2B4, as breast cancer progressed. Conversely, there was an upregulation of the inhibitory receptor NKG2A, which diminishes NK cell cytotoxic function and evasion of NK cell-mediated anti-tumor immunity (197, 198). Another study revealed a decline in the levels of NKp46, a lysis receptor responsible for direct tumor cell elimination, within the TME compared to normal cells (199). Immunotherapies targeting NK cells encompass various strategies to improve their activity, including promoting ADCC with mABs (200), blocking inhibitory signals (201), utilizing cytokines to augment NK cell proliferation and cytotoxicity through CAR NKs (202), IL-15 (203), and adoptive transfer of NK cells (204). In recent years, adoptive cell therapy strategies have emerged as a promising approach for utilizing NK cells (205). These immunotherapies entail the isolation, activation, and expansion of immune cells, which are then reintroduced into patients to combat tumor cells. A noteworthy application of this technique involves equipping NK cells with cancer-targeting CARs (206). However, the potential of engineered NK cells is hindered by immunometabolism limitations caused by factors such as hypoxia and cytokine stimulation in the TME (194, 207). Further studies are needed to understand how NK cell immunometabolism in TME regulates their anti-tumor properties.

### Lymphoid cells in breast TME

#### T- lymphocytes

Tumor-infiltrating lymphocytes (TILs) in TME regulate the induction of robust anti-tumor immunity, immunosuppression, efficacy of ICB therapy, cancer metastasis, and resistance to novel combinational ICB therapies (208). The TILs found in the TME primarily consist of CTLs, B cells, NK T cells, and CD4^+^ T helper cell subsets, including IFN-γ-producing CD4^+^ (Th1) cells, IL-4-producing CD4^+^ (Th2) cells, Foxp3^+^CD4^+^ regulatory T cells (Tregs) (209). Recent advancements in sub-type classification of TILs, using techniques such as flow cytometry, genomic approaches (single-cell RNA-seq, 10X genomic sequencing), and ICB therapies targeting T cells, have resulted in an increased emphasis on identifying TILs and potential immunological prognostic biomarkers specific to different subtypes of breast cancer (210, 211). Despite the ability of Th1 and CTLs to stimulate strong anti-tumor immunity, the TME employs various immune evasion strategies to suppress the infiltration, activation, and effector functions of CTLs and Th1 cells, inhibiting host anti-tumor effector responses. One extensively studied mechanism involved in this process is the upregulation of inhibitory receptors on T cells and higher expression of inhibitory ligands by tumor cells and APCs within the TME (212). APCs define the T cell differentiation and activation through tumor antigen presentation on MHC molecules to T cell receptors (TCR), expression of CD80 and CD86 ligands, which bind to co-receptors (such as CD28, ICOS, PD-1, CTLA4), and secretion of specific cytokines that define the fate of T cell differentiation (213). These co-signaling receptors can stimulate or inhibit T cell activation and effector functions. Examples of inhibitory receptors include PD-1, CTLA-4, LAG3, and TIM-3 (214). These receptors are crucial in maintaining immune balance and preventing excessive T cell activation during infections. However, tumors highly promote the expression of co-inhibitory receptors on T cells in TME to promote immune evasion. PD-1, for instance, binds to PD-L1 or PD-L2 ligands expressed by various immune cells or cancer cells to facilitate immune evasion (214). PD-1 possesses an inhibitory immunoreceptor tyrosine-based inhibition motif (ITIM) and immunoreceptor tyrosine-based switch (ITSM) motif in its cytoplasmic tail (215). When T cells engage with tumor cells and APCs, PD-L1 phosphorylates ITIM/ITSM, resulting in the recruitment of TCR-phosphorylating kinase, cytosolic tyrosine phosphatases (SHP-1 and SHP-2), and the inhibitory tyrosine kinase (216). As a result, the PI3K/Akt and Ras/MEK/Erk pathways necessary for initiating T cell activation are weakened. Recent research has shown the potential of blocking the PD-1/SHP-2 interaction as a novel approach to PD-1 inhibition (217). Accordingly, several monoclonal antibodies (mAbs) targeting PD-1 (pembrolizumab and nivolumab) and PD-L1 (atezolimumab) interaction have received FDA approval for the treatment of various lethal cancers including metastatic melanoma, Hodgkin’s lymphoma, head and neck squamous cell carcinoma, and breast cancer, among others (218).

In breast TME, infiltrating T cells demonstrate an upregulation of PD-1, while APCs (DCs and macrophages) and tumor cells exhibit higher expression of PD-L1 (219). The expression of PD-1 on CD4^+^ TILs is correlated with the invasiveness of breast cancer (220). Moreover, recent studies have shown decreased CD4^+^ and CD8^+^ T lymphocyte infiltration in DCIS and IDC breast cancer subtypes (221). These findings suggest that the reduced number of T lymphocytes in TME contributes to the transition of TNBC and HER2^+^ cancer subtypes from DCIS to IDC, resulting in a poor prognosis and worsened overall survival (OS) (222). Another recent study revealed the efficacy of CD3-HAC, a bifunctional fusion protein engineered to target EA1-mesenchymal stromal cells against metastatic breast cancer (223). CD3-HAC specifically binds to PD-L1-positive tumor cells to attenuate the impact of PD-1/PD-L1 on T cells exposed to MDA-MB-231, leading to enhanced T cell activation and stimulated lymphocyte-mediated lysis both *in vitro* and *in vivo* (223). In addition to immune evasion, the heightened expression of PD-1 on T cells indicates T cell exhaustion. CD8^+^ T cell exhaustion was initially identified in mice infected with chronic lymphocytic choriomeningitis virus (LCMV) infection (224). In this condition, the chronic presence of viral antigens constantly activates and stimulates CD8^+^ T cells, resulting in a decline in their effector functions (224, 225). In the TME, immune cells experience continuous stimulation from tumor antigens (226). Consequently, their metabolism and transcription profile change, ultimately leading to functional exhaustion (227). Immune cell exhaustion in TME is characterized by persistent tumor antigens stimulation, reduced proliferation capacity, enhanced inhibitory receptor expression, and decreased production of effector cytokines such as IL-2, TNFα, or IFN-γ (228).

In a comprehensive cohort study of breast cancer patients, it was discovered that despite the prevalence of T lymphocytes in IDCs, a significant portion of T cells exhibited reduced activity or were inactive due to exhaustion. These exhausted T cells displayed heightened expression of co-inhibitory receptors, PD-1 and CTLA-4, and diminished levels of active anti-tumor T cell subsets, CD62-L and CD127 (229). Phenotyping and functional analysis studies unveiled a distinctive T cell differentiation subset associated with exhaustion (230). It was observed that the underlying transcriptional mechanisms differed between effector T cells and exhausted T cells (231). This distinction was reflected in the expression of phenotypic markers, with effector CD8^+^ T cells exhibiting high levels of CD44 and killer cell lectin-like receptor subfamily G member 1 (KLRG1), while exhausted T cells displayed low or intermediate levels of these markers (232). Conversely, inhibitory receptor markers were highly expressed on exhausted T cells compared to effector CD8^+^ T cells (231). Additionally, exhausted T cells exhibited disparate expression of the transcription factors EOMES and T-bet, whereas effector CD8^+^ T cells expressed both simultaneously (233). The TME plays a critical role in inducing functional exhaustion in CD8^+^ T cells by promoting the cell surface expression of CD39, an immunosuppressive molecule (234). CD39^+^CD8^+^ T lymphocytes displayed an exhausted phenotype characterized by reduced production of IFNγ, TNF-α, and IL-2 and increased expression of co-inhibitory receptors such as PD-1 and CTLA-4. Targeting CD39^+^ appears promising in restoring T cell function and as a potential therapeutic intervention (234, 235). Revitalization of exhausted CD8^+^ T lymphocytes can be achieved through the inhibition of PD-1:PD-L1 interaction (236), CTLA-4 (237), and LAG-3 (238). Clinical studies that block the PD-1/PDL-1 inhibitory pathway to restore CD8^+^ T cell ability to proliferate and carry out its cytotoxic functions have been reported in other cancers. For instance, while pembrolizumab and atezolizumab are effective PD-1/PDL-1 inhibitors in second-line advanced non-small cell lung cancer (NSCLC), avelumab and durvalumab were effective in late-phase clinical testing (239). Another clinical study proposed donor lymphocyte infusion (DLI) targeting T cell exhaustion in hematology malignancy (240). In a clinical study, their findings reveal that patients who received DLI had a significant increase in CD8 cell counts, while the levels of CD4 T cells and B cells remained unaffected, indicating the potential of DLI to reverse CD8^+^ T cell exhaustion (241). However, the use of DLI alongside other T cell exhaustion revitalization methods has been suggested (242). While research on T cell exhaustion in breast cancer subtypes remains limited, future investigations aimed at revitalizing exhausted T cells and enhancing active T lymphocyte proliferation hold immense potential for the development of safe and effective immunotherapies against breast cancer.

The presence of regulatory T lymphocytes (Tregs), specifically the Foxp3 expressing subtype, is associated with a negative prognosis in breast cancer patients (243). Tregs express co-inhibitory receptors such as PDL-1, CTLA-4, and PD-1, which promote local immunosuppression and contribute to the spread of breast cancer (244, 245). Targeting Tregs can lead to a breakthrough in immunotherapy. Current strategies developed to inhibit Tregs’ harmful impact in the TME include inhibiting their recruitment, favoring their transformation into effector CD4^+^ T-cell subsets, blocking their expansion, depleting Tregs, and impeding their suppressive function (246). Further research and clinical trials are needed to fully understand the dynamics of T cell exhaustion and explore the use of combination therapies that can enhance T cells’ effector and cytotoxic functions.

#### B- lymphocytes

B lymphocytes are primary mediators of humoral immunity. In the induction of adaptive immunity, B cells stimulated by antigens, along with the assistance of helper T cells, undergo differentiation into antibody-secreting plasma cells, initiating adaptive immune responses (247). In tumors, B lymphocytes are commonly found in the lymph nodes and invasive margins (248). Their impact on tumor onset and progression can be positive, negative, or passive (249). Upon activation by antigens, B cells undergo differentiation into antibody-secreting plasma cells. There are five subtypes of human immunoglobulins (Ig): IgG, IgA, IgM, IgD, and IgE (250). Among these five Ig types, IgG accounts for approximately 75% of the antibodies found in human serum (251). Despite being highly preserved, IgG is classified into four, namely IgG1 – IgG4, which exhibit varying effector functions based on their interaction with Fcγ receptors (FcγR) (252). Activation of FcγR-expressing cells triggers ADCC and phagocytosis of tumor cells (253). Conversely, when expressed by tumors, IgG-FcγR interaction can promote tumor progression (254, 255). A study conducted by Ma et al. revealed an abundance of IgG-expressing cancer cells in 68 breast cancer cases, encompassing 40 primary cancers and 28 metastatic cancers (256). Their findings demonstrated that IgG-expressing breast cancer cells exhibit more aggressive biological behavior, indicating the progression and metastasis of breast cancer. Moreover, the formation of circulating immune complexes (CICs) from the Ag-Ab complex can activate FcγR on myeloid cells, leading to the generation of MDSCs (257). These MDSCs effectively suppress the anti-tumor function of CD4^+^ and CD8^+^ T cells (42). B cells actively induce tumor cell apoptosis by producing granzyme B, a potent cytolytic molecule (258). These granzyme B-producing B cells can perform vital effector and regulatory functions during immune responses (258). Notably, granzyme B derived from carcinoma sources has been observed to effectively eliminate tumor cells *in vitro (* 259). However, it is worth noting that the presence of granzyme B in breast tumor tissue can degrade the TCR-zeta subunit in the TCR, thereby impeding TCR assembly, expression, and anti-tumor signaling. This phenomenon occurs particularly in continuous antigen exposure and chronic inflammation (260). Moreover, the production of cytokines such as IL-2, IL-4, IL-6, IL-7, IFN-γ, IFN-α, TNF-α, CCL7, and CCL28 can stimulate an anti-tumor response (261, 262). These vital molecules are crucial in B cell maturation, differentiation, and survival (261). Notably, CCL28 and CCL27 direct the migration of plasma cells to mucosal sites during breast cancer anti-tumor response, correlating with improved prognosis (263). Conversely, other chemokines produced by B cells like CCL5, CCL20, and CCL1 are known to attract TAMs, Tregs, and MDSCs and induce EMT in breast cancer cell (264).

The immunosuppressive B cell subtype, Bregs, produce IL-10, IL-35, IL-15, and TGF-β cytokines that suppress CD8^+^ T-cell cytotoxicity, Treg recruitment, and M2/Th2 polarization (265–268). A study with a mouse 4T1 model of breast cancer demonstrated that the secretion of IL-10 by B lymphocytes acts in a TGF-β-dependent manner to promote the conversion of naive CD4^+^ T cells to Foxp3^+^ Tregs (269). Also, a chemokine, CXCL13, functions to recruit B cells to TME, where they differentiate into Bregs and stimulate EMT in tumor cells (270). A study showed that nano-trapping CXCL13 reduces Bregs differentiation, leading to prolonged cancer-free survival (271). Bregs-specific phenotype PD-1-PD-L1^+^CD19^+^ has been reported to exert the greatest suppressive effects on T effector cells (272). Two separate groups, Campbell et al. and Miligy et al. in 2017, revealed that B cells with phenotypes CD19^+^, CD24^+^, and CD38^+^ were correlated with increased tumor proliferation and risk of recurrence in breast cancer subtypes ER^-^, PR^-^ and HER2^+^ (273, 274). The findings from their studies also suggested that CD20^+^ is a prognostic marker for better patient outcomes. Conversely, numerous studies have shown that Foxp3^+^ Tregs also express CD20^+^ and can be indicative of poor prognosis in breast cancer (273–277). Hence, conducting in-depth research to accurately define and differentiate the CD20^+^ anti-tumor role in B cells and the pro-tumor role in T cells is necessary.

### Stromal cells in breast TME

#### Cancer-associated fibroblasts

CAFs are heterogenous cells that demonstrate their significance in various aspects of breast cancer, including growth, metastasis, response to treatment, and resistance to anti-cancer therapies (278). These cells derive from a range of sources, including normal fibroblasts, myofibroblasts, mesenchymal cells, stellate cells, fibrocytes, pericytes, smooth muscle cells, preadipocytes, or bone marrow-derived cells (279). Additionally, recent research by Flores et al. has identified CD34^+^ stromal cells/telocytes as another origin of CAFs, particularly in the invasive lobular carcinoma (ILC) subtype (280). Throughout tumor progression, CAFs contribute to the production of crucial structural proteins like elastin and collagen type I-V, which are involved in basement membrane formation (281), inflammation (282), epithelial differentiation (283), and angiogenesis (284). Moreover, CAFs produce MMPs, which are responsible for the degradation of the ECM and play a role in ECM homeostasis (285). Increased proliferation and secretion of growth factors, immunomodulatory factors, and ECM proteins have also been observed in CAFs and linked to their role in breast cancer (286). These CAF-specific markers can be used to identify breast cancer biomarkers and hold significant importance in diagnosis, prognosis, and the development of novel therapeutic approaches against breast cancer (287, 288). CAF biomarkers are not exclusive to CAFs, thereby requiring a comprehensive characterization to accurately define CAFs. Notably, biomarkers such as α-SMA, vimentin, desmin, cadherin-11, integrin α1β1, and MMPs are utilized for identifying CAFs originating from myofibroblast (286). However, there remains a lack of clear understanding regarding the detailed characterization of pro-tumor phenotypes of CAFs and their associated biomarkers. Initial studies employing SNP array analyses, multi-gene sequencing, and whole exome sequencing have reported the absence of somatic mutations in CAF phenotypes (289, 290). Subsequent findings have suggested somatic mutations and loss of heterozygosity as indicative of CAFs in the tumor stroma (291, 292). Furthermore, additional reports have demonstrated that epigenetic modifications, such as DNA methylation, may be responsible for maintaining the CAF phenotype and contributing to cancer cell growth and progression (293, 294). Hence, further studies are required to precisely characterize the pro-tumor properties of CAFs.

CAFs secrete growth factors such as TGF-β, EGF, FGF-2, TNF-α, platelet-derived growth factor (PGDF), and VEGF-A, and express cell surface and extracellular matrix proteins (295, 296). Extensive research links CAFs to breast cancer progression, with studies showing that CAFs secrete SDF-1/CXCL12 and HGF, both of which promote breast cancer growth and metastasis (297). HGF activates c-Met on tumor cells, leading to enhanced metastasis, while SDF-1 facilitates tumor growth and angiogenesis through the CXCR4 receptor on breast carcinoma cells. These functions promote the transition of breast carcinoma from ductal carcinoma *in situ* (DCIS) to invasive ductal carcinoma (IDC) (297). Recent targeting of HGF/c-Met interaction has emerged as a significant breakthrough in breast cancer therapy (298). Additionally, SDF-1 secretion by breast CAFs contributes to the proliferation of breast cancer stem cells (CD44^+^CD24^-^) and the induction of drug resistance (299, 300). Therefore, targeting SDF-1 holds great promise for breast cancer therapeutics. Furthermore, CAFs play a crucial role in immune evasion by regulating the miR-92/PD-L1 pathway during breast cancer progression (301, 302). Molecular profiling of CAFs in breast tissue and carcinoma has identified differentially expressed genes (DEGs) that can serve as diagnostic and prognostic biomarkers and be targeted for developing new therapies (303, 304). Notably, high PDGF expression by CAFs indicates a shorter median survival for breast cancer patients (305).

**Figure 3:**
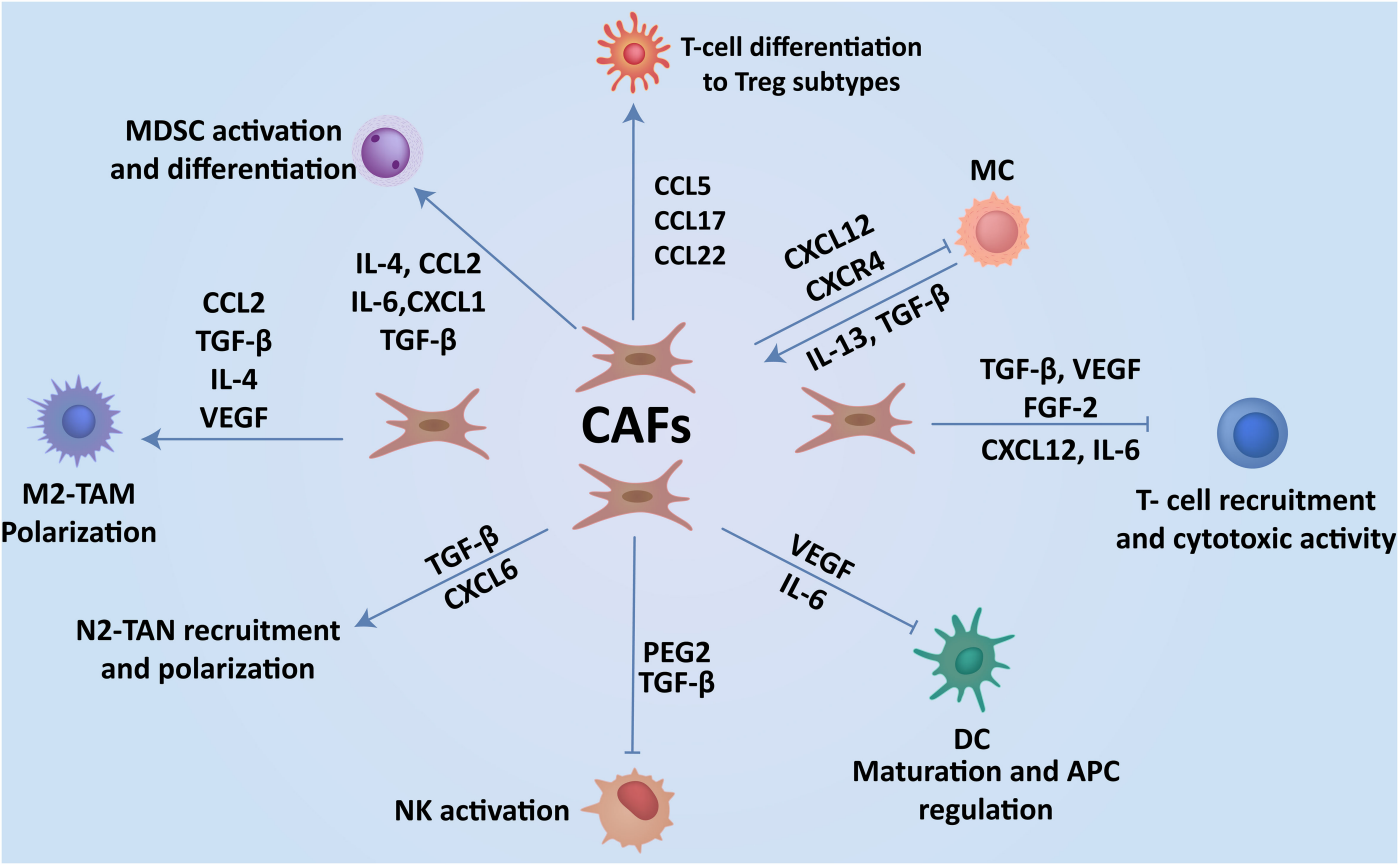
CAFs-immune cell interplay contributes to breast cancer progression. Interaction of CAFs with immune cells via the production of cytokines and soluble factors create an immunosuppressive TME, which enhances the progression of cancer to metastasis.

Numerous oncogenic and immune cell signaling pathways within the TME cross-regulate CAFs and immune cells (**Figure 3**), promoting tumor progression, immunosuppression, and drug resistance (306). These pathways encompass TGF-β/Smad, Wnt/β-catenin, EGFR, TGF-β, PI3k/AKT/mTOR, JAK/STAT3, etc (307). Shangguan et al. previously demonstrated that inhibiting the TGF-β/Smad signaling pathway in human bone marrow mesenchymal cells hinders their differentiation into CAFs (308). Additionally, suppressing the EGFR signaling pathway, a crucial factor in EbbB/HER subtype metastasis has shown potential to inhibit CAF-associated cancer stemness (309). Therapies targeting CAFs have proved effective in overcoming treatment resistance in HER2^+^ breast cancer, with increased expression of NK-IL2RS, NK, and NKT cell signatures before treatment correlating with improved response to anti-HER2 mAbs-based therapy (310). Therapeutic targeting of CAF signaling pathways within the TME presents a promising approach for achieving breast cancer remission. Considering the significant role of CAFs in breast cancer metastasis and the complexity of cancer cell molecular signatures (311), further research and clinical trials are imperative to establish their potential utility in breast cancer prognosis and therapeutic intervention.

#### Cancer-associated adipocytes

CAAs are adipocytes that actively reside near cancer cells, promoting crucial communication by releasing factors that can induce localized and systemic effects (312). Adipocytes in the TME can change in response to signals from cancer cells, leading to the formation of CAAs. These CAAs may release fatty acids into the surrounding tissue which can be taken up by breast tumor cells (313). The increased demand for energy and building blocks for rapidly dividing cancer cells makes fatty acids a valuable substrate for their metabolic needs. Within the TME, fatty acids undergo β-oxidation and serve as the principal source of ATP which promotes tumor survival and proliferation (314). Breast cancer cells can utilize fatty acid oxidation (FAO) as a metabolic pathway to oxidize fatty acids and generate energy. This process becomes particularly relevant in situations where other energy sources, such as glucose, are limited. Enhanced fatty acid metabolism, including FAO, has been associated with increased tumor aggressiveness in breast cancer (315). Fatty acids not only serve as an energy source but also play a role in various signaling pathways that can influence cell survival, proliferation, and invasiveness. Fatty acids can also activate specific lipid signaling pathways within tumor cells leading to changes in gene expression and metabolic pathways (316, 317). For instance, fatty acids can activate peroxisome proliferator-activated receptors (PPARs) beside other nuclear receptors, which can regulate genes involved in lipid metabolism, inflammation, and cell growth (318). PPARs are a group of nuclear receptors that play a crucial role in the regulation of fatty acid metabolism and energy homeostasis (319). Activation of PPARs occurs when ligands, such as fatty acids or their derivatives, bind to the receptors. Once activated, PPARs form heterodimers with retinoid X receptors (RXRs) and bind to specific DNA sequences called PPAR response elements (PPREs) in the promoters of target genes (320). This binding regulates the transcription of genes involved in lipid metabolism, energy homeostasis, and inflammation (321). In hormone receptor-positive breast cancer, estrogen receptor-positive (ER+) tumors can be influenced by adipose tissue-derived factors. Fatty acids and other adipokines may affect the growth and behavior of ER+ breast cancer cells (322). Although all adipose depots can secrete inflammatory factors, such as TNF, IL-6, IL-1β, and TGF-β (323) obese visceral adipose primarily releases excessive fatty acids, cholesterol, triglycerides, hormones, and adipokines, closely associated with metabolic dysfunction and unfavorable cancer outcomes (324). Additionally, adipocytes can contribute to chemotherapeutic drug resistance, as their co-culture with fibroblasts can deactivate the effectiveness of anti-cancer drugs by metabolizing them into less potent secondary metabolites (325). Understanding the metabolic interactions between CAAs and breast cancer cells, specifically involving fatty acids and FAO, has implications for developing targeted therapies. Researchers are exploring ways to disrupt these metabolic pathways as potential strategies to inhibit tumor growth and improve treatment outcomes.

Furthermore, TME’s plasticity allows for transdifferentiation, a process whereby cells undergo a significant shift in their identity, thereby acquiring new transcriptional or morphological characteristics typical of a different cell lineage. Microenvironmental cues including neighboring cells, extracellular matrix, blood vessels, and immune cells can induce shifts in cancer cell phenotypes (326). Emerging research has shown that cancer cells, can exhibit plasticity and undergo transdifferentiation, which can contribute to tumor heterogeneity and complicate treatment strategies (327). Despite these challenges, researchers are exploring ways to harness lineage plasticity for therapeutic purposes. In a research conducted by Ronen et al, to capitalize on the plasticity of cancer cells, breast cancer cell differentiation was redirected towards a non-malignant and non-proliferative adipocyte fate (328). In this study, the utilization of Rosiglitazone and an MEK inhibitor as part of the therapy appears to be particularly effective against aggressive characteristics of breast cancer cells, consequently inhibiting metastasis. The transdifferentiated adipogenesis-induced cancer cells, MTDECad and 3T3-L1 cells formed become functional post-mitotic adipocytes which have comparable characteristics with functional adipocytes. For instance, both differentiated cell types express the adipocyte-specific markers C/EPBa, PPARg2, and fatty acid binding protein 4 (FABP4), and they secrete the adipocyte-specific adipokine adiponectin (328). Therapeutic strategies need to consider the evolving nature of cancer cells and the potential for phenotypic changes under different microenvironmental conditions. Generally, CAA significantly influences various aspects of breast cancer, including risk, progression, migration, metastasis, and resistance to existing treatments (329). Therefore, targeting the interaction between adipose tissue and breast cancer may be a promising approach to overcoming immune tolerance and drug resistance.

### Vasculature cells in breast TME

#### Endothelial cells

ECs are a constitutive part of the cardiovascular system and are critical to homeostasis, angiogenesis, and immune response (330). They regulate the passage of substances through tight cell junctions and line the basement membrane of capillaries (331). ECs, along with a basal lamina and strategically positioned pericytes, form the structure of blood vessel walls (332). ECs facilitate intravasation, allowing cancer cells to migrate into the blood vessel lumen, a critical step in cancer metastasis (333). Tumor growth relies on a blood supply, and during rapid growth, tumors stimulate neovascularization by weakening the basement membrane of existing blood vessels (334). Upon the secretion of angiogenic factors like VEGF-A, PDGF, hypoxia-inducing factors (HIF-1), and MMPs, the basement membrane degrades. This basement membrane degradation triggers the migration of endothelial cells and pericytes to the tumor region, contributing to TME angiogenesis (334). Additionally, tumor-associated hypoxia, mediated by HIF-1α and HIF-2α, plays a role in malignant conversion and metastasis, as well as influencing immune cell functions within the TME (335). Schneider & Miller’s study revealed that angiogenesis precedes the progression of mammary hyperplasia to malignancy in breast cancer (336). They demonstrated that transfection of tumor cells with angiogenic stimulatory peptides promoted tumor growth, invasiveness, and metastasis (337). Clinical outcomes have substantiated the efficacy of anti-angiogenic therapy as a viable treatment approach. However, the use of antiangiogenic drugs in conjunction with conventional chemotherapy in metastatic breast cancer has shown limited clinical impact on overall survival (337). It is essential to conduct further studies by addressing potential obstacles, such as toxicity, drug resistance, and alternative angiogenesis mechanisms, in order to optimize the effectiveness of anti-angiogenic therapies in breast cancer progression.

Recently, correlation between neurogenesis and angiogenesis in breast TME has been linked to aggressive breast cancer breast cancer (338). Tumors release neurotrophic factors that can initiate innervation, a process that imitates angiogenesis (339). Hence, tumor neurogenesis is intricately linked to metastasis, as the presence of ingrown nerve endings can release neurotransmitters that significantly enhance the development of metastatic cells (339). In an immunohistochemistry analysis of carcinoma breast tissues, it was observed that protein gene product (PGP) 9.5 protein was present in 61% of IDC tissues compared to fibroadenoma and DCIS, particularly in ER-negative and node-negative subtypes (340). PGP 9.5, a ubiquitin-carboxyl hydrolase, is an enzyme expressed throughout the stages of differentiation in nerve tissue of mice brains and is a useful marker for detecting central nervous system damage (341). Likewise, in both ER-negative and node-negative subgroups of breast IDC, a significant association was observed between PGP 9.5 expression and higher microvessel density (MVD), compared to less expression of PGP 9.5 and MVD identified in DCIS. The analysis reveals a clear correlation between neurogenesis and angiogenesis, particularly in ER-negative and node-negative subtypes of breast cancer (340). In a human breast cancer cohort study, a significant association between neurogenesis, consolidated neuro-angiogenic signature, and high-grade breast cancer features was observed (342). Single cell-based spatial mapping with imaging mass cytometry was used to identify the colocalization of neural and vascular structures, indicating the presence of neurovascular niches within tumor tissue. Cancer cells can release various signaling molecules, including growth factors and cytokines, that play a role in recruiting both sprouting axons (microaxons) and endothelial cells (microvessels) to the TME (343). This phenomenon is referred to as neurotropism, and it has been observed in several types of cancers, including breast cancer (343, 344). The exact mechanisms by which cancer cells influence axon recruitment are still an area of active research. The coexistence and potential coregulation of microaxons and microvessels suggest a complex interplay between neural and vascular elements within the tumor stroma.

#### Pericytes

Pericytes are mural cells that envelop blood vessels and reside adjacent to the endothelial cells lining the capillaries. Pericytes play a crucial role in the development and stabilization of the vasculature through TGF-β signaling activation (345). Also, pericytes actively enhance the physical stability and support of endothelial tubule function during the initial phase of angiogenesis by co-occupying endothelial tubules (346). Within the TME, the tumor vasculature serves multiple functions, such as supporting tumor growth and facilitating metastasis to distant organ sites (347). Notably, breast cancer is a highly vascularized tumor with extensive pericyte coverage (347). Targeting angiogenesis during breast cancer progression can be approached by inhibiting the vessel-stabilizing properties of vascular pericytes (348, 349). Depleting pericytes has been shown to increase intra-tumoral hypoxia and lung metastasis in advanced-stage hypoxic tumors with pre-established vasculature (348). The presence of perfusion defects in breast cancer blood vessels is associated with vessel dilation, tortuosity, and inadequate perivascular coverage (347, 350). This abnormal vascular system is partly attributed to morphological and molecular alterations in pericytes and significant population heterogeneity (347). The presence of pericytes in the primary TME impedes cancer progression and metastasis (350). Distinguishing pericytes can be achieved through morphological characteristics and molecular markers, including α-SMA, desmin, PDGFR-β, CD248, NG2, and angiopoietin-2 (349, 351). Many of the pericyte markers are used in several studies to calculate the mean microvascular pericyte coverage index (MPI). For instance, α-SMA expression in breast cancer yielded an estimated MPI range of 32%-80%. Other markers such as NG2, PDGFRβ, desmin, and CD248 have also been employed for MPI measurement (351). Other markers such as NG2, PDGFRβ, desmin, and CD248 have also been applied in MPI measurement (350). Many anti-angiogenic treatments involve targeting endothelial cells or proangiogenic factors to suppress neovascularization cause tumor cell death. In the context of anti-angiogenic treatments, simultaneously targeting both endothelial cells and pericytes has been suggested (352). According to some studies, non-selective elimination of pericytes may not provide benefits but may instead promote tumor aggressiveness and metastasis. Therefore, gaining a comprehensive understanding of pericyte heterogeneity in response to changes in the TME can inform effective pericyte targeting strategies (351, 353).

## Conclusion and future directions

The current research findings on the interaction between the TME and cancer cells have significantly advanced our understanding of their crucial roles in cancer progression and treatment response. Traditionally, treatment strategies for breast cancer predominantly focused on promoting tumor cell death. However, the emergence of immunotherapy has revolutionized cancer treatment by incorporating anti-tumor immune responses and targeting TME cells. Successful research of some clinical trials targeting breast TME has provided a promising outlook for utilizing these cells in cancer therapies (**Table 2**). The cells within the breast TME can either act against or promote tumor cells; in certain conditions, they may exhibit dual roles. The ability of TME cells to switch from anti-tumor to pro-tumor functions poses a significant challenge for immunotherapy. The anti-tumor and pro-tumor functions of these cells primarily depend on specific mediators such as cytokines, chemokines, and growth factors in TME. The interplay between these mediators generated by the cellular components modulates the TME towards either an anti-tumor or an immunosuppressive environment. Despite the initial findings on breast TME, future studies should focus on understanding the evasion of anti-tumor immunity and exploiting TME cell mediators to target cancer cells. Immunotherapy has emerged as a critical component in the treatment of various types of cancer, including breast cancer.

ICB therapies have shown remarkable efficacy when used alone or in combination with other treatment modalities. Reprogramming CTLs through ICB immunotherapies has been successful, but the resistance caused by TAMs to recently developed ICB therapies remains challenging. Therefore, there is a need for targeted inhibition of TAMs to enhance tumor cell-killing capacity, as well as further investigation into repolarizing TAMs towards an anti-tumor phenotype. Moreover, the emerging field of immunometabolism and understanding how TME regulates metabolism in immune cells to suppress anti-tumor immunity is crucial to developing novel immunotherapies and overcoming resistance against conventional and ICB therapies. The potential of adoptive immunotherapy, specifically equipping NK cells with cancer-targeting CARs, is hindered by immunometabolism. Extensive research is required to understand how regulating the metabolism of NK and other immune cells in TME can promote their anti-tumor activities. Furthermore, there is a pressing need for further investigation into the recovery of exhausted T-cells and NK cells to promote their effector functions. The complexity and heterogeneity of TME cells, such as the CAFs and pericytes, present challenges in their proper characterization. The recent advances in next-generation sequencing, metabolomics, and bioinformatics, which study cancer progression at both tissue and single-cell levels, can be employed to identify novel breast cancer stage-specific biomarkers, functional phenotype of immune and non-immune cells in TME, resistance to cancer therapies, and development of novel targeted immunotherapies. Such investigations will lead to improved stage-specific breast cancer diagnosis, the development of innovative TME cell-specific targeted immunotherapies with fewer side effects, and overall improved quality of life and survival for women with highly metastatic breast cancer.

## Author contributions

TA: Conceptualization, Writing – original draft, Writing – review & editing. RM: Writing – original draft. AA: Writing – original draft. SP: Writing – review & editing. PM: Writing – review & editing. LA: Writing – review & editing. AS: Writing – original draft, Writing – review & editing.
